# Cyber Dating and Sexual Fantasies in Men Who Have Sex with Men from Spain: Analyzing the Intersection of Technology, Intimate Relationships, and Violence on *Scruff*

**DOI:** 10.1007/s10508-026-03417-1

**Published:** 2026-04-15

**Authors:** Álvarez-Domínguez Pablo, Antonio García-Gómez

**Affiliations:** 1 Faculty of Education Sciences, Department of Theory and History of Education and Social Pedagogy, University of Seville, Seville, Spain; 2 Faculty of Philosophy, Department of Modern Philology, University of Alcalá, 28801 Madrid, Spain

**Keywords:** Rape culture, Femininities, Sexual scripts, Technology-facilitated violence, Sexual orientation, *Scruff*

## Abstract

Prior research on rape culture has largely focused on heterosexual women and has underexamined men who have sex with men (MSM) in digital contexts and there is little work on midlife users of *Scruff*. This study addresses these gaps by analyzing how sexual scripts on *Scruff* intersect with intimacy, gendered positioning, and technology facilitated sexual violence among Spanish MSM. By using a mixed methods discourse analysis, we examined 300 profiles posted by men aged 40 to 50 years who reside in the Madrid region of Spain. The analysis identified three recurring clusters of scripts. First, many profiles staged feminized self-presentations that commodified femininity and aligned passivity with desirability. Second, rape themed fantasies framed male sexual entitlement as exciting and largely unproblematic. Third, dominant top identities assumed control and initiative while constructing feminized partners as available and violable. Together, these patterns show how enactments of masculinity and femininity are closely tied to the authorization of sexual aggression and the downplaying of harm, and how femmephobia reworks heteronormative gender hierarchies within MSM spaces. At the same time, some profiles invite readings that emphasize consensual kink or playful exaggeration, underscoring the interpretive complexity of online profiles. The study contributes to debates on masculinities, platform mediated intimacies, and technology facilitated sexual violence by documenting how MSM sexual scripts reiterate gendered power asymmetries. Implications include the need for consent focused sexual health education tailored to MSM.

## Introduction

A burgeoning body of literature has examined the normalization of abuse in digital sexual image exchange (e.g., Naezer, 2020), alongside persistent attention to traditional sexual double standards of promiscuity and virginity (Ashley et al., 2022). These double standards encourage men to seek sexual conquests and assert dominance, while women are expected to be chaste and submissive, a discrepancy that research shows can fuel sexual aggression (Jamshed et al., 2022). Yet feminist research on sexual aggression and rape culture has largely focused on young heterosexual women (Kessel, 2022; Klement et al., 2022), leaving a notable gap regarding mediated intimacies and sexual harms experienced by men who have sex with men (MSM) in digital contexts. This gap includes limited attention to how MSM construct sexual subjectivity (a sense of oneself as a sexual being) and sexual agency (the capacity to make autonomous choices about sex).

To analyze how these forms of sexual subjectivity and agency are patterned and constrained, we draw on sexual script theory. Sexual scripts are socially shared frameworks that organize how people understand, enact and evaluate sexual behavior. Classic accounts distinguish among cultural scenarios (dominant narratives about who should do what, with whom, and under what conditions), interpersonal scripts (recurrent interactional patterns in specific encounters), and intrapsychic scripts (individual fantasies, desires and self‑understandings) (Simon & Gagnon, 1986). Together, these layers shape what is perceived as “normal,” desirable or dangerous sex, structure expectations around consent and risk, and position subjects in relation to power, pleasure and vulnerability.

This gap is especially pressing given the rapid rise of online dating platforms, which have transformed how people, including MSM, seek intimate connections, arrange dates and negotiate sexual encounters (Parry et al., 2024). These platforms create new opportunities for connection but also distinct risks. Research shows that MSM who use dating apps are at elevated risk of sexual violence compared to their heterosexual peers, with a significant proportion reporting unwanted sexual experiences initiated through these platforms (Henry & Powell, 2016). At the same time, key features of these platforms (such as anonymity, visual self‑presentation and algorithmic matching) can obscure risk cues and complicate consent negotiations, creating specific challenges for MSM navigating digital intimacy (Aitken & Taylor, 2024). Unlike in heterosexual contexts, where gendered scripts often assign sexual agency and risk along binary lines, MSM must negotiate agency, desire and vulnerability within a framework where both partners may be subject to, and agents of, sexualized power.

While the literature on abuse and hook‑up culture in heterosexual contexts provides a useful foundation, the ways these dynamics unfold among MSM, how they perceive risk, negotiate consent and experience or perpetrate sexual harm, remain understudied and warrant focused attention. A critical analysis of MSM’s use of dating platforms must therefore account for the specific configurations of sexual subjectivity and agency that emerge in same‑sex male encounters, and for how gendered assumptions and experiences of oppression are both reproduced and contested in digital spaces. In what follows, we review research on the performance of masculinities and femininities in digital environments, with particular attention to dating platforms used by MSM. This review foregrounds the persistent hegemony of patriarchal masculinity and the systemic devaluation of femininity, including through phenomena such as femmephobia (Hoskin, 2017), and highlights how masculinity, sexuality and violence intersect and are intensified online.

### Performing Masculinities/Femininities in Online Spaces

Connell’s (1995) theorization of hegemonic masculinity remains central for understanding how masculinities are organized as a hierarchy that privileges some forms while subordinating others, including femininities and non-hegemonic masculinities. Hegemonic masculinity refers to a culturally exalted way of being a man that legitimizes men’s collective dominance over women and other masculinities; it is less a description of most men’s lives than a normative ideal that shapes institutions, expectations, and everyday interactions. Rather than treating masculinity as a fixed identity, this framework presents it as a dynamic regulatory force that polices gender boundaries and reinforces systemic inequalities. Subsequent scholarship shows that hegemonic masculinity also operates within same sex male contexts. Although gay men are typically positioned as a subordinated masculinity within the broader gender order, many align themselves with key elements of hegemonic masculinity, for example by valuing sexual dominance or straight acting styles (Bridges & Pascoe, 2014; Connell, 1992). This work demonstrates that hegemonic masculinity functions as a wider normative template that can be reproduced, negotiated, or resisted within gay male communities.

In MSM communities, both partners occupy the position of men within the gender order, yet hierarchies of masculinity persist through the policing of femininity and the valorization of hypermasculine presentations (Connell, 1992; Rodriguez & Hernandez, 2018). Sexual agency and subjectivity are negotiated not along male or female lines but through the performance and regulation of masculinity and femininity within same sex interactions. Building on this, gender scholarship emphasizes that gender is performative, produced through repeated acts rather than expressing a fixed inner essence, and that it is relational and intersectional, recognizing masculinities and femininities as plural, fluid, and embedded in sociocultural power relations (Connell & Messerschmidt, 2005; Schippers, 2007). Hoskin (2019, 2024) calls for close attention to the complexities of occupying feminine positions within patriarchal discourses, showing how femininity is both resisted and reproduced within masculinist frameworks.

The rise of digital technologies has opened new pathways for investigating these dynamics. Work on social media and self-presentation shows that online environments play a central role in identity construction, as users curate their profiles in response to platform affordances and audience feedback (Gündüz, 2017). Digital affordances such as anonymity, visual self- presentation, and algorithmic curation enable experimentation with gendered identities but can also reinforce existing hierarchies and exclusions (Aitken & Taylor, 2024). Research on gay dating apps indicates that MSM both reproduce hegemonic masculinity and exercise sexual agency in ways that differ from heterosexual contexts (Rodriguez & Hernandez, 2018). The literature on mascing shows how users emphasize their own masculinity and seek masculine partners, creating a masculine elite that mirrors heteronormative hierarchies even in spaces largely free from direct homophobia.

### Masculinity, Sexuality, and Violence: Intersecting Discourses in Digital Contexts

The intersection of masculinity, sexuality, and violence is a critical area of inquiry, especially as these dynamics are increasingly mediated through digital technologies (de Simões et al., 2024). Research on digitally mediated domination, such as financial domination communities on Twitter, shows how social media architectures script and circulate asymmetric power relations and contested negotiations of consent (Gündüz et al., 2023). In same sex male contexts these scripts operate differently from heterosexual ones. Without the binary of male aggressor and female victim, sexual agency and vulnerability are distributed and negotiated within encounters where both partners may occupy dominant or subordinate positions. This shift does not remove gendered power dynamics; instead, femininity, whether performed by MSM or ascribed to certain roles, becomes the devalued position against which masculine agency is asserted. MSM who perform or are associated with femininity may therefore experience both sexual objectification and exclusion from sexual agency, mirroring dynamics of oppression found in heterosexual contexts but through different mechanisms (Rodriguez & Hernandez, 2018).

Dominant discourses of masculinity often celebrate aggression, dominance, and sexual entitlement, which can normalize harmful behaviors about male sexuality (Russell et al., 2025). On dating apps, these dynamics intensify as self-presentation and interactional scripts valorize male sexual assertiveness and construct femininity as passive, compliant, or violable (Featherstone et al., 2024). From a sexual script perspective, hegemonic masculinity functions not only as an identity ideal but also as a set of expectations that script men as active initiators of sex and femininity as available and violable. These cultural scenarios are translated into interpersonal scripts on dating apps, where users anticipate and rehearse particular sequences of sexual interaction. For MSM, such scripts are reworked within same sex encounters yet continue to organize who is expected to initiate sex, who is framed as available or violable, and whose boundaries are more easily ignored.

Ging (2019) argues that narratives of masculinity are embedded within wider cultural frameworks that valorize aggression, complicating how men negotiate their identities. The tension between in group and out group values heightens these challenges, as men navigate dominant narratives while grappling with marginalized social positions (Roig-Mora et al., 2025). For MSM, this dual positioning as marginalized gay men and potentially privileged men shapes how sexual subjectivity and agency are constructed, with implications for both perpetration and victimization of sexual harm (Russell, 2021). This body of work underscores the need to examine how masculinities and femininities are performed within LGBTIQ+ communities and how digital platforms participate in reproducing gendered power structures and sexual violence.

### Femmephobia: The Systemic Devaluation of Femininity

Integral to understanding masculinities is a critical engagement with the systemic devaluation of femininity, conceptualized as femmephobia. Drawing on Hoskin (2017), femmephobia captures how femininity is routinely denigrated in patriarchal societies, as empirical work shows that masculine traits are culturally valorized while femininity is marginalized in ways that shape both subjectivities and broader gender ideologies (Allan, 2025; Bhatti, 2022). Hoskin’s typology distinguishes several interrelated forms of feminine devaluation. Perceived femmephobia is rooted in internalized misogyny and leads women and others who embody femininity to devalue themselves and one another, including through seemingly benign or patronizing attitudes that help trivialize and normalize sexual violence and harassment (Dietzel, 2021, 2024; Schwartz, 2022). Ascribed femmephobia works through linguistic and discursive practices that legitimize some femininities while delegitimizing others, reinforcing hegemonic masculinity and marginalizing men who are feminized for failing to meet dominant masculine ideals (Kastrup et al., 2023). Related concepts such as femme mystification and pious femmephobia highlight how femininity is commodified, morally policed, and subordinated to masculinity, naturalizing male dominance, discrediting feminine embodiment practices, and sustaining rape culture (Hopner & Chamberlain, 2020; Menzie, 2022).

These forms of femmephobia expose failures to protect femininity both within and beyond LGBTIQ communities (Hoskin et al., 2024) and provide a framework for understanding how occupying male and or female positions is linked to the endorsement of sexual harm scripts (Hearn et al., 2025). In MSM contexts, femmephobia operates as a central mechanism through which gendered power hierarchies are reproduced even in the absence of women. Drawing on sexual script theory, we use sexual harm scripts to refer to recurrent patterns in cultural and interpersonal scripts that normalize, excuse, or trivialize coercive or non-consensual sexual practices, defining who is presumed always willing, whose refusals are negotiable, and whose violations are dismissed. In MSM digital settings, these scripts are closely tied to femmephobic hierarchies, as feminized positions are repeatedly cast as disposable, perpetually available, and less entitled to safety.

### The Current Study

The present study centers gender hierarchy (Kincaid, 2022; Schippers, 2007), hegemonic masculinities (Connell, 1987, 2003; Messerschmidt, 2019), and feminine identity (Hoskin & Whiley, 2023) to examine how men who have sex with men construct and navigate sexual and gender identities in digital contexts. Drawing on Critical Femininities (Dahl, 2012; Hoskin & Blair, 2022), it explores how gender hegemony shapes masculine and feminine practices within LGBTIQ+ spaces. More specifically, it analyzes how MSM on dating apps discursively produce and negotiate masculinity and femininity by positioning themselves in relation to feminine coded behaviors, language, and presentations, what we term occupying the social location of woman in Hoskin’s (2024) sense. Social location here refers to how individuals embody and perform femininity while negotiating the marginalization and devaluation attached to feminine identities (Hoskin, 2017). For MSM, this may involve adopting feminine pronouns or aesthetics in profiles, using language that feminizes sexual roles, such as references to being used or treated like women, and experiencing femmephobia when feminine traits are associated with lower social and sexual desirability (Steele et al., 2024).

The study focuses on how sexual subjectivity and agency are constructed and constrained through these positionings. MSM who perform masculinity may access greater agency and desirability, while those who occupy feminized positions may face diminished agency, objectification, and heightened vulnerability to sexual harm. These dynamics parallel but do not replicate those in heterosexual contexts. By foregrounding them, the study clarifies how gender assumptions and experiences of oppression operate differently in same sex male relationships while revealing shared patriarchal logics that devalue femininity across contexts. It shows how gender hierarchies and the regulation of femininity are both reproduced and contested within digital intimate spaces used by MSM.

Using discourse analysis of Spanish MSM profiles on *Scruff*, the study offers a qualitative and quantitative examination of narratives of technology facilitated sexual harm and violence. In line with sexual script theory, self-presentation strategies and profile texts are treated as sites where cultural scenarios around masculinity, femininity, and sexual violence are translated into interpersonal and intrapsychic scripts. *Scruff* profiles and interactions are understood as places where MSM articulate, reproduce, and contest expectations about who should desire whom, who is expected to be sexually available, and how consent, refusal, and risk are navigated. Building on Dietzel’s (2024) claim that rape culture is usually read through a heteronormative lens that leaves MSM implicated but not recognized as affected, the study addresses the need for a more nuanced understanding of MSM experiences of sexual violence in digital environments. It examines the sexual scripts that emerge in profiles and narratives, how they distribute agency and vulnerability, and how they normalize or problematize technology facilitated harms (Patel & Roesch, 2022).

Central to the analysis is how sexual agency and subjectivity shape and are shaped by experiences of safety, risk, and harm. The study investigates how MSM negotiate consent, desire, and vulnerability in ways that both echo and depart from heterosexual norms, recognizing that assumptions about male aggression and female passivity do not simply transfer to same sex encounters. Instead, agency and vulnerability are allocated according to performances of masculinity and femininity, so that those in feminized positions face heightened risk (Colliver, 2023). Overall, the study explores sexual norms and practices within MSM dating apps, contributing to debates on rape culture and digitally mediated intimacies by showing how sexual scripts and media driven sexual socialization (Wright, 2011) structure gay men’s use of dating and hook up apps.

## Method

### Participants

*Scruff*, alongside *Grindr* and *Wapo*, is among the most widely used gay dating apps in Spain. *Scruff* offers three primary features: (1) it allows users to filter profiles based on various criteria such as age, weight, height, preferences, and community affiliations (i.e., “tribes”); (2) it provides the option to mark profiles as “favorites” or block specific users when necessary; and (3) it enables the sharing of voice notes, photographs, short videos, and location data, alongside text messages and stickers, while also allowing users to input detailed personal information. Although *Scruff* facilitates socializing and the formation of friendships, its primary function is to enable users to seek out dates, sexual encounters, or romantic relationships (Whitfield et al., 2017). In essence, the predominant motivation for its users is to engage in sexual encounters (Thompson, 2018).

While all three platforms offer location-based services that allow users to view nearby profiles and interact through actions such as "tapping" or "woofing" on profiles, or by sending direct messages, *Scruff* targets a more specific demographic. As Ostheimer and Iqbal (2019) observe, *Scruff* attracts a wider age range and is notably preferred by more mature gay individuals, with a smaller representation of transgender users. Most research on gay dating apps has focused predominantly on Grindr and its younger user base, leaving more mature men underrepresented in the existing literature (Queiroz et al., 2023). This lack of attention to middle‑aged gay men on these apps represents, on the one hand, a gap in understanding how these men navigate relationships, sexual encounters, and social connections and, on the other, a missed opportunity to shed light on the specific needs, desires, and challenges of older gay men in an increasingly digitalized world (Morris, 2023). Addressing this gap can provide a more nuanced view of LGBTIQ+ online spaces.

### Measures and Procedure

This study examined user profiles on *Scruff*, a location‑based hook‑up application, between April 2024 and November 2024. The analytic dataset comprised 300 user profiles, all extracted directly from the *Scruff* platform. We selected these profiles purposively rather than randomly, based on the inclusion criteria detailed below, and they are therefore not intended to be statistically representative of all *Scruff* users. The sample focused on male users aged 40–50 years, allowing for a nuanced exploration of linguistic practices and social interactions within a specific age cohort. As noted by Marciano and Nimrod (2021), studying middle aged men is particularly relevant due to the relative stability of their identities, in contrast to younger men, who are often in a phase of exploration and experimentation.

Moreover, *Scruff's* location-based framework, which connects users with others in their immediate geographic area, ensured that all 300 profiles in this dataset belonged to men residing in the region of Madrid (Spain). Given the regional context, all profiles were originally written in Spanish. After we produced the initial English translations, two linguists (bilingual speakers of Spanish and English, with training in linguistics) independently reviewed them, focusing in particular on slang, sexual terminology, and culturally specific expressions. We flagged segments that involved ambiguous or highly colloquial language, including potentially reclaimed slurs, and discussed them until we reached consensus on an English rendering that preserved not only the propositional meaning but also the register, evaluative stance, and degree of offensiveness. For especially salient or contested items, we carried out targeted back translation checks, asking one of the linguists to retranslate the English version into Spanish to ensure that no major meaning drift had occurred.

To explore the presence of technology facilitated narratives of sexual violence within this context, we selected profiles based on the following criteria: (1) inclusion of information on age, physical characteristics, and personality traits; (2) explicit statements of sexual preferences, detailing users’ desires and boundaries; (3) descriptions of sexual interests and expectations; and (4) explicit references to sexual aggression, including physical and verbal aggression.^1^ Importantly, the inclusion criterion of explicit references to sexual aggression captured both rape-themed fantasies and descriptions that could, in principle, correspond to coercive, non-consensual acts. The profile texts themselves do not provide access to offline interactions, partners’ perspectives, or negotiation processes, and some users explicitly frame their descriptions as “fantasies” or “role play” scenarios. Accordingly, throughout this article, we distinguish analytically between rape-themed fantasy (that is, scenarios in which aggression and the suspension of consent are eroticized in the text and may or may not be enacted consensually in practice) and sexual harm scripts (recurrent linguistic patterns that normalize, excuse, or trivialize the waiver of consent).

The coding and analysis therefore operate at the level of textual representation rather than attempting to classify particular authors or encounters as definitively consensual or non-consensual in offline contexts. All 300 profiles in the analytic dataset met criteria (1) through (4). This constitutes a purposive sub‑corpus of aggression‑themed profiles, constructed to allow in‑depth discourse analysis of how sexual aggression is articulated and negotiated in MSM digital self‑presentation. Because the total number of *Scruff* profiles of MSM aged 40–50 in Madrid during the sampling period is unknown, the dataset is not used to estimate the overall prevalence of aggression on the platform. Rather, we selected these inclusion criteria to provide a focused basis for examining the construction of sexual and gendered identities in profiles where aggression is made explicitly salient, as well as the ways users navigate and present their boundaries and preferences within the digital environment.

The analysis of the 300 profiles yielded a corpus of 23,974 utterances (32,425 tokens). In this study, we use utterance to refer to the smallest stretch of text that realizes a single communicative act (e.g., “Top only,” “Safe sex always,” “Come and play”), which may consist of a single word, a short phrase, or a full sentence. Tokens refer to individual orthographic words in the corpus, after basic cleaning (for example, removing duplicated punctuation and standardizing abbreviations).

Coding was carried out at the segment level (utterance‑by‑utterance), not at the profile level: each profile typically contained multiple utterances, reflecting the highly elliptical and list‑like register that characterizes many *Scruff* self‑presentations (e.g., strings of descriptors separated by line breaks or commas). Drawing on Tsui’s (1994) functional model of discourse acts, which distinguishes between eliciting acts (moves that seek information, confirmation, or commitment), directive acts (moves that attempt to get the addressee to do something), and informative acts (moves that provide evaluation), we developed a typology of communication acts for this dataset. This typology comprises three higher‑order types of communication acts (eliciting, directive, and informative acts), which are further subdivided into eight specific subtypes tailored to the *Scruff* corpus. Our aim was not to replicate Tsui’s model in full, but to adapt its core distinctions in order to describe the recurring patterns and regularities that emerged from the data.

- **Eliciting acts**: (1) Elicit: confirm a particular piece of information (e.g., “Are you interested in trying something new tonight?”); and (2) Elicit: commit to a course of action (e.g., “Will you take a chance on a fun night out with me and your cock?”).
- **Directive acts**: (3) Directive: suggestion of a course of action (e.g., “You should be mounting my juicy pussy from behind and having a wild night together”); (4) Directive: threat (e.g., “Don't stare at my trouser department, or you will awaken the beast and I’ll have to rape your filthy juicy pussy”; (5) Directive: imposition of a course of action (e.g., “Come and rape my arse!).
- **Informative acts**: (6) Informative: (in-)direct positive appraisal of the profile user (e.g., “I have the most impressive clit on Earth”); (7) Informative: (in-)direct positive appraisal of the prospective date (e.g., “Just real men like you who can ride me hard can have me”); (8) Informative: (in-)direct negative appraisal of the prospective date (e.g., “With a tiny dick, you won’t last me five minutes”).

We developed the coding scheme and codebook through a combined deductive and inductive process, with both authors working together at each stage. We began from Tsui’s (1994) tripartite distinction between eliciting, directive, and informative acts, and from the literature on sexual scripts and sexual harm scripts, and applied these preliminary categories to an initial subset of profiles (approximately 10 to 15 percent of the dataset). During this pilot phase, we identified recurrent patterns that were not fully captured by the initial labels, for example, differences between threats and other directives, or between appraisals of the self and of the prospective date, and refined the categories accordingly. For each of the eight subtypes, we then wrote an operational definition, inclusion and exclusion criteria, and decision rules specifying how to code utterances that could plausibly fit more than one category. The resulting codebook also includes several example utterances for each subtype, and a condensed version of these categories and examples is presented above. Once the full corpus had been coded using this Tsui-informed scheme, we aggregated the codes to produce the quantitative summaries reported in Tables 1 through 3, which show how often each discourse-act subtype occurred in the corpus and within each thematic subset of profiles.

**Table 1:** Commodification of femininity: Distribution of discourse acts

		Freq	Ratio
Elicitations	confirm	432	4.07
	commit	627	5.91
Directives	threat	1489	14.04
	imposition of a course of action	2045	19.28
	suggestion of a course of action	96	0.90
Informatives	(in-)direct positive appraisal of the profile user	2263	21.34
	(in-)direct positive appraisal of the prospective date	3535	33.33
	(in-)direct negative appraisal of the prospective date	116	1.09
	Total	10,603	utterances

N = number of utterances in this thematic subset coded with the given discourse-act subtype. Percentages refer to the proportion of all utterances in this subset (Table 1: N = 10,603 utterances across 161 profiles; Table 2: N = 8,162 utterances across 142 profiles; Table 3: N = 5,209 utterances across 97 profiles). Due to rounding, column percentages may not always sum to exactly 100%

In relation to consent, the coding distinguished between the fantasy frame of a profile and the specific utterances through which consent, refusal, or its suspension were linguistically scripted. Utterances such as “I never say no” or “Don’t ask for permission” (example 7), even when embedded in profiles that explicitly marked the content as fantasy, role play, or kink, were coded according to their communicative function within the text. For instance, these utterances were treated as directives that preempt a partners request for permission or relocate the responsibility for setting limits. Explicit meta‑pragmatic markers such as “fantasy,” “role play,” or references to kink communities were recorded in analytic memos to contextualize the reading of these utterances, but no separate category was created to label profiles as “consensual” or “non‑consensual” at the level of offline practice, as the data do not allow such inferences. The distinction developed in the Discussion is therefore between consensual rape‑themed fantasies (as they may be lived by participants) and sexual harm scripts as recurring linguistic patterns that discursively normalize the waiver of consent, irrespective of whether any given encounter would, in practice, be negotiated as consensual.

Once the codebook was stabilized, we applied it to the full set of 23,974 utterances. We carried out coding manually. Both authors coded the data. We initially double coded a substantial subset of utterances independently to check for consistency, discussed all discrepancies, and refined the wording of definitions and decision rules until we reached agreement. After this calibration phase, we divided the remaining material and continued to code independently while meeting regularly to review difficult or ambiguous cases. We assigned each utterance a single primary code corresponding to its most salient communicative function as defined in the codebook. When an utterance appeared to instantiate more than one type, for example, combining evaluation and a directive, we followed the decision rules to determine the primary category and recorded any secondary reading in analytic memos.

To enhance accuracy and consistency, we refined the codebook iteratively during the pilot phase, maintained a written audit trail documenting coding decisions, category refinements, and difficult cases, and engaged in periodic peer debriefing with a colleague experienced in discourse analytic research, who reviewed the evolving codebook and a sample of coded excerpts and discussed borderline or discrepant cases. In line with qualitative best practice, we adopted these procedures in place of formal interrater reliability measures to support the transparency, credibility, and dependability of the coding.

Analytically, we combined a corpus informed quantitative description of communication acts with close, context sensitive qualitative readings of profile texts. After coding all 23,974 utterances using the eight discourse-act subtypes described above, we generated descriptive statistics for the corpus as a whole and for each of the three thematic subsets of profiles discussed in the Results section. For Tables 1, 2 and 3, we calculated the absolute frequency (N) of each discourse-act subtype and its relative frequency, expressed as the percentage of all utterances in the relevant subset (reported in the “Ratio” column). We then used these quantitative summaries to identify especially salient configurations, for example, the high proportion of threats and imposed directives in the rape fantasy and dominant top subsets, which in turn guided our selection of illustrative excerpts and informed the subsequent qualitative interpretation of how sexual harm scripts are discursively realized.

**Table 2:** Femininity and rape-themed fantasies: Distribution of discourse acts

		Freq	Ratio
Elicitations	confirm	643	7.87
	commit	297	3.63
Directives	threat	1765	21.62
	imposition of a course of action	1926	23.59
	suggestion of a course of action	25	0.30
Informatives	(in-)direct positive appraisal of the profile user	1146	14.04
	(in-)direct positive appraisal of the prospective date	1893	23.19
	(in-)direct negative appraisal of the prospective date	467	5.72
	Total	8,162	utterances

**Table 3:** Dominant top scripts in rape-themed fantasies: Distribution of discourse acts

		Freq	Ratio
Elicitations	confirm	69	1.32
	commit	26	6.25
Directives	threat	1334	25.60
	imposition of a course of action	933	17.94
	suggestion of a course of action	38	0.72
Informatives	(in-)direct positive appraisal of the profile user	574	11.01
	(in-)direct positive appraisal of the prospective date	655	12.57
	(in-)direct negative appraisal of the prospective date	1280	24.57
	Total	5,209	utterances

### Analytical and Ethical Position

In designing and conducting this study, we followed current guidance on internet research ethics and data protection, particularly for research using user‑generated content in online environments. *Scruff* is a login‑gated application, and the data analyzed here derive from profiles that users chose to make visible to any logged‑in user within their local area. We accessed only material that was available to us as ordinary users within the application and did not use automated scraping tools, circumvent access restrictions, or attempt to view profiles outside our demographic or geographic scope. We did not seek individual informed consent because the study relied solely on naturally occurring profile texts that users had already made available within the platform, and we de-identified all data at the point of collection in order to minimize privacy risks.

To minimize the risk of identification, we recorded only the textual self‑descriptions from profiles and did not store usernames, profile photos, exact GPS coordinates, or other direct identifiers. We assigned each profile a numerical code at the point of transcription, and we stored the working dataset on a password protected, encrypted drive accessible only to us. Furthermore, we acknowledge that the expectations and social norms surrounding online dating are likely reflective of broader socially accepted sexual norms and practices (Fileborn, 2016). In line with the theoretical framework outlined above, our analysis focuses on how users’ profile texts express and negotiate sexual scripts and norms surrounding aggression, consent, and risk in MSM digital encounters.

Discourses surrounding male sexual drive and sexual violence are not new. Rather, as Bogetić (2021) argues, the extensive reach of media driven sexual socialization not only sustains these narratives but actively promotes them. In Bogetić’s (2021, p. 117) words, “dating apps do not merely construct friendly spaces for expressing non-normative desire, but they largely perpetuate many of the (locally and globally current) normative restraints.” The present study does not seek to stigmatize or pathologize individuals who fantasize and/or engage in consensual aggressive sexual activity.

However, we treat descriptions of consensual, non consensual, and coercive encounters as narratives that aim to validate the users sexual prowess and entice others into sexual engagement. Specifically, the discourse analysis of these scripts serves three primary objectives: (1) to explore the dominant sociosexual norms within this online environment; (2) to enhance understanding of MSM’s sexualities, mediated intimacies, and perceptions of risk; and (3) to interrogate whether the sexual behaviors and norms they fantasize about may participate in the normalization, condoning, or trivialization of sexual violence at the level of discourse.

Given the purposive nature of the dataset, all of which consists of profiles that explicitly reference sexual aggression, these analyses do not aim to quantify how common such content is on *Scruff* as a whole. Claims about the “normalization” or “prevalence” of aggression should therefore be understood as referring to the discursive patterns within this aggression‑themed corpus, rather than to all profiles of MSM aged 40–50 on the platform. This approach prioritizes depth of analysis of sexual harm scripts in contexts where they are overtly articulated, while acknowledging the limits of generalizability.

## Results

Analysis of the 300 *Scruff* profiles identified three main sets of recurring sexual scripts. First, a large group of profiles used feminized self-presentations that foregrounded availability and receptivity, often positioning the author as occupying a feminine or “female” role vis-à-vis a more masculine partner. Second, a substantial subset of profiles combined feminized self-positioning with explicit rape-themed fantasies, describing scenarios of being “raped”, overpowered, or used “without mercy.” Third, another subset positioned the author as an active “top” or dominant man who enacts aggression on a partner framed as submissive or feminized. In what follows, we describe each of these patterns in turn, with indicative quantitative distributions of discourse acts and illustrative excerpts from profiles.

### “Use Me as You Please”: Exploring the Commodification of Femininity

Within the sample of 300 profiles, 161 men, approximately 53.7 percent, adopted a feminized position through self disclosure and self promotion. They did so through explicit self referential language that positioned themselves as women, the use of feminine pronouns in Spanish, and descriptions of their bodies using terminology conventionally associated with female embodiment, for example, I am your female. At the same time, they positioned prospective partners within a masculine framework, frequently describing them as dominant figures, for example, female passive looking for alpha fucker. This dual positioning illustrates how gendered identities are negotiated in *Scruff* profiles, with masculinity and femininity performed as relational roles. Table 1 summarizes the distribution of discourse acts in this subset:

As shown in Table 1, informative acts that positively appraise the prospective date, 33.3 percent of utterances, and the profile user, 21.3 percent, are particularly frequent, alongside directives that impose a course of action, 19.3 percent. In this subset, users most often describe and evaluate themselves and their desired partners while also issuing non-negotiable invitations or demands. The qualitative excerpts below illustrate how these patterns are realized.

Across these profiles, users construct a highly sexualized self by foregrounding availability, repeated references to sex with multiple partners, and self-positioning as throwaway or disposable. They describe themselves as insatiable, refer to having been with many alpha fuckers, and present themselves as holes for multiple partners, framing their capacity to be penetrated and used as the primary basis of their desirability. In doing so, they occupy what we term the social location of woman, aligning themselves with feminized roles while positioning interlocutors as decisively masculine, dominant figures. The following examples illustrate this pattern:

(1) 39 years old. Profile pic: Completely naked, in bed on all fours.

Marica insaciable. Pluma. Mi cama siempre está caliente. Han pasado muchos maxos ¿quieres ser el siguiente? Usa mi coño, de usar y tirar. [Insatiable faggot. Effeminate. My bed is always hot. I have slept with many alpha fuckers. Do you want to be next one? Use my cunt, a throwaway cunt]

(2) 41 years old: Profile pic: Completely naked, in the shower.

Pasiva. Deportista. Tenerte en mi cama y dejarte hacerme, mi hobi favorito. Mi cuerpo es tuyo, úsalo pa correrte. 24/7 [Female passive. Sportive. Having you in my bed and letting you do me is my favorite hobby. My body is yours, use it to cum. 24/7]

In example (1), the user presents himself as “insatiable,” foregounds effeminacy, and invites the reader to “use my cunt, a throwaway cunt,” casting himself as a disposable sexual resource rather than a reciprocal partner. The accumulation of descriptors (insatiable, effeminate) constructs a subjectivity defined by limitless availability and by being repeatedly taken by masculinized others. In example (2), the user self identifies as “female passive” and describes his body as the property of the prospective partner (“My body is yours, use it to cum”), naturalizing an asymmetrical distribution of agency in which his role is to be continually available for another man’s pleasure. Similar formulations appear in other profiles:

(3) 40 years old. Profile pic: Wearing female underwear.

Tal y como ves en la foto. Soy un agujero que le siembren un buen rabo como el tuyo. Soy super pasiva 100%. Busco maxo. Soy tu hembra. Mi xixi necesita de un lefador insaciable. Pluma máxima. 24/7 [As you see in the picture. I’m a hole to seed a big cock like yours. I’m 100% power bottom. Looking for alpha fucker. I’m your female. My cunt needs a cock who cums non-stop. Super effeminate. 24/7]

(4) 42 years old. Profile pic: Naked chest, holding a banana.

Pasiva busca macho. Alta. Mona.. Culo prieto. Se siente solo. Ven a hacerle compañía. Me derrito con maxos como tu. Hazme tuya y te daré las gracias…[Female passive looking for alpha fucker. Tall. Pretty A tight bottom that feels alone. Come and keep my bottom company. I melt with men like you. Do me and I’ll thank you for that]

In both examples (3) and (4), the user adopts explicitly feminized labels (“super passive,” “female passive”), foregrounds specific bodily attributes such as tight bottom, and constructs the imagined partner as the decisive alpha fucker who sets the terms of the encounter. Metaphors of being “a hole to seed a big cock” and of a lonely “tight bottom” waiting to be “made” someone’s own combine feminized identity claims, affective vulnerability, and sexual receptivity.

Across these profiles, users present themselves as highly available and receptive partners, foregrounding passivity as their main attraction. In examples (1)-(4), this is done through self labeling and through metaphors that reduce the self to a sexualized body or orifice. Users explicitly invite the reader to use them and frame their desirability in terms of constant availability and openness to multiple partners. In each case, the user positions himself as the feminized, receptive party and casts prospective partners as masculine figures, linking feminized self‑presentation, bodily availability, and the search for dominant men as key elements in this subset of profiles.

### “Rape me without Mercy”: Femininity and the Eroticization of Rape-Themed Fantasies

Within the sample, 142 profiles, 47.3 percent, adopted a feminine position while explicitly centering rape themed or aggression themed sexual fantasies. In these profiles, users describe themselves as passive objects of desire and frame prospective partners as dominant agents who rape, hurt, or destroy them. Many descriptions include explicit references to violence and minimize or suspend refusal. Table 2 summarizes the distribution of discourse acts in this subset:

Directives of imposition and threat are especially salient in this subset; together they account for almost half of all utterances, 23.6 percent and 21.6 percent respectively, indicating that the anticipation or description of aggression is central to how desire is articulated. Users couple feminized self positioning with invitations to be overpowered, injured, or raped, and repeatedly relocate the work of boundary setting to the imagined partner. The following examples illustrate how feminized identities are interwoven with rape themed fantasies and the preemptive suspension of consent:

(5) 44 years old. Profile picture: Naked chest, Licking a finger.

Busco pollon que mi viole sin compasión. Me cabe toda y todas. Marica pasiva busca maxo, maxo. Tu pones los límites. No me tiens que drogar, arrancame ropa y destrozame [Looking for a horse hung that rapes me with no mercy. I can take it all. I can take any cock. Bottom looking alpha fucker. You set the limits. You don’t have to drug me, rip off my clothes and smash my arse with your cock]

(6) 39 years old. Profile picture: Completely naked in bed.

Hembra sumisa x macho. Nunca diga no. Tu mandas. Te recibo de rodillas a cuatro. Pide y te lo haré. Arrancame la virginidad [Submissive female looking for an alpha man. I never say no. You are the boss. Fuck me on all fours. Just tell me and I’ll do it as you say]

(7) 35 years old. Profile picture: Completely naked, on all fours.

Pasiva de las buenas. No pidas permiso. No duele. Goza. Taladra como quieras, fuerte, ostias, sangre, goza. [A classic bottom. Don’t ask for permission. It doesn’t hurt. Just enjoy. Drill me as you wish, ride me hard, slapping, blood, enjoy]

In example 5, the user explicitly requests to be raped with no mercy, asserts his capacity to take it all, and delegates responsibility for limits to the other man, you set the limits. This combination of feminized self description, as bottom, and the invitation to have clothes ripped off and to be smashed constructs a scenario in which the only active agent is the masculinized partner, while the self is positioned as a compliant object of aggression. Example 6 similarly presents the user as a submissive female who never says no, greets the partner on all fours, and accepts that you are the boss, thereby naturalizing permanent availability and the abdication of refusal. In example 7, the user goes further by instructing the partner do not ask for permission, denying the relevance of pain, it does not hurt, just enjoy, and listing intense acts, such as slapping and blood, as desirable components of the encounter. Other profiles intensify this pattern by combining feminized self labeling with references to female body parts and more elaborate fantasies:

(8) 36 years old. Profile pic: Wearing a woman’s thong in bed.

Casi virgen. Busco hombre k me viole. Yo tu putita. Tanga, medias y taconazo. Vestida o desnuda. Sexo fuerte. Entrar a saco. Insultos, puños, insultos, lluvias doradas. [Almost a virgin. Looking for a man that rapes me. I’m your slut. Thong, stockings and super high heels. Dressed or naked. Strong sex. Insults, fists, insults, golden showers]

(9) 43 years old. Profile pic: Semen stained face.

Chocho vicioso. Obligame a comer tu nabo, lamer tus huevos y sobacos. No quiero amor, solo sexo. Un clitoris desinibido que taladrar. [Vicious cunt. Force me to eat your cock, lick your balls and armpits. No love, just sex. Uninhibited clit to be drilled].

In examples 8 and 9, the users construct explicitly female coded personae by naming themselves your slut, vicious cunt, and uninhibited clit to be drilled, and by referring to clothing and accessories conventionally associated with femininity, such as a woman’s thong, stockings, and very high heels. Both profiles explicitly invite the partner to rape or force them and list concrete acts and bodily positions, such as dressed or naked and force me to eat your cock, lick your balls and armpits. The focus is on being acted upon, being raped, forced, drilled, while the prospective partner is implicitly positioned as the agent who decides what will be done and how far it will go.

Taken together, examples 5 through 9 show a consistent configuration in which the profile user occupies a feminized, passive role and invites a more masculine partner to enact rape themed or otherwise aggressive scenarios. Across these profiles, users describe themselves as submissive females and refer to female coded body parts and accessories to mark their position within the encounter. At the same time, they explicitly call for the partner to rape, force, or drill them or to rip off their clothes, often coupled with statements that downplay the possibility of refusal. In this subset, rape themed expressions, instructions that minimize or suspend consent, and references to feminized bodies are regularly combined with positive descriptions of the imagined partner and with imperatives or invitations to carry out aggressive acts.

### “I Rape You, Kick You Out of the Car, and Leave You Dead in the Gutter”: Dominance and Consent in Rape-Themed Fantasies

Within the sample of 300 profiles, 97 men, 32.3 percent, explicitly positioned themselves as active top gay men, consistently using male pronouns to assert their social location as men. Unlike the previous group, these users presented themselves as agents and initiators of sexual aggression, narrating rape scripts in which they dominate and control their prospective partners. These scripts presume that partners will share and consent to the fantasy of being overpowered and raped by a dominant man. In doing so, they reinforce norms that valorize masculinity as active and controlling and femininity as submissive and receptive. Table 3 summarizes the distribution of discourse acts in this subset:

Threats and negative appraisals of the prospective date are particularly frequent, together comprising over half of all utterances in this subset, 25.6 percent and 24.6 percent respectively, and reflecting a focus on control, punishment, and degradation in the described encounters. The following examples illustrate these patterns:

(10) 38 years old. Profile pic: Completely naked and dildo in his hand.

Busco maricona a mucha honra. Quiero agarrarte, tirarte del pelo y follarte el ojete sin compasión. Rabazos, dildo, puños. Busco puta de usar y tirar [Looking for Flaming faggot. I want to take you, grab you by the hair, and fuck you hard in the arse. Long dick strokes, dildos, fists. Looking for a throwaway slut]

In (10), the user presents himself as the sole active agent in the encounter, stating that he will “take” the partner, “grab [him] by the hair,” and “fuck [him] hard.” The sequence “long dick strokes, dildos, fists” lists a progression of increasingly intense penetrative acts. The addressee is named with explicitly demeaning terms, signaling that they are envisaged as an anonymous, disposable object for use rather than as an equal partner. In (11), the user focuses on ejaculation and impregnation as the central elements of the desired scenario. The text positions the user as the one who decides when and how to ejaculate and attributes a transforming power to his cum, while the partner is portrayed as a passive recipient who merits this treatment.

(11) 41 years old. Profile pic: Naked semen-stained chest.

Me da morbo preñar sin avisar. Te lo mereces por zorra. Sentir cómo mi lefa te hace mujer. [Breeding you makes me excited. You deserve it because you’re a slut. Feeling how my cum makes you a real woman]

Example (12) explicitly excludes affection (“no snogging”), narrowing the encounter to “full‑on rough” sex. The user lists a series of violent acts (“tear your filthy arse apart,” “I’ll stomp on your neck…”) and specifies that he alone will decide their timing and intensity. He tells the partner that if they cannot take it they should not make contact and states that he will not stop till he is done, instructing them instead to scream like a faggot and take my pounding. The partner is addressed with feminizing and insulting terms, bitch and faggot, and is expected to endure whatever the author chooses to do.

(12) 43 year old. Profile pic: Completely naked in bed.

Nada de besos. Rabazos a saco para desgarrarte el ojete de puta. Ostias, pisarte el cuello como me plazca. Si no lo aguantas, no me llames. Hasta que no me corra no paro, grita cual marica y aguanta mis folladas [No snogging, mate. Just going full-on rough to tear your filthy arse apart, you bitch. Proper clouts, I’ll stomp on your neck whenever I feel like it. If you can’t take it, don’t bother calling me. I’m not stopping till I’m done, scream like a faggot and take my pounding]

Taken together, examples 10 through 12 depict profiles in which the author presents himself as the doer of aggression and assigns the partner a purely receptive, degraded role. In each case, the user narrates what he will do, taking, grabbing, and so on, while framing the other man as a flaming faggot, slut, bitch, or faggot who is there to be used, punished, or made into a real woman. Affection and reciprocity are absent or explicitly ruled out, and the limits of the encounter are set unilaterally by the user. Across this subset, directives that impose specific actions, threats of pain or injury, and negative appraisals of the prospective partner are tightly interwoven. Dominance, control over pace and boundaries, and indifference to the partners potential resistance are presented as core features of the desired sexual script.

## Discussion

Analysis of the 300 *Scruff* profiles authored by MSM aged 40 to 50 in Madrid identified three partially overlapping sets of recurring sexual scripts. Of the 300 profiles, 161 featured feminized self-presentations that foregrounded availability and receptivity, often positioning the author as occupying a feminine or “female” role in relation to a more masculine partner.

A substantial subset of 142 profiles combined feminized self-positioning with explicit rape themed fantasies, describing scenarios of being “raped,” overpowered, or used “without mercy.” Another 97 profiles positioned the author as an active “top” or dominant man who enacts aggression on a partner framed as submissive or feminized. In this section, we interpret these patterns in relation to sexual script theory (Simon & Gagnon, 1986), hegemonic and plural masculinities (Connell, 1995, 2003; Connell & Messerschmidt, 2005), femmephobia (Hoskin, 2017, 2019, 2024), and research on technology facilitated sexual violence (Dietzel, 2021, 2024; Patel & Roesch, 2022). We argue that, taken together, these patterns exemplify what we term sexual harm scripts: recurrent ways of imagining and narrating sex that make coercion, the suspension of consent, and the devaluation of feminized partners appear normal, arousing, or inconsequential.

The conceptual framework for this discussion can be summarized as follows. From existing scholarship, the analysis draws on (1) hegemonic and plural masculinities as a gender order that privileges certain ways of being “real men” and marginalizes feminized subjects; (2) Hoskin’s typology of femmephobia as a set of mechanisms through which femininity is devalued, policed, and punished; and (3) research on technology facilitated sexual violence that conceptualizes digital platforms as sites where everyday communicative practices can enact and normalize sexual aggression.

The present study contributes, in turn, the notion of sexual harm scripts and shows how they are realized in this MSM *Scruff* corpus through specific patterns of digital speech acts, including clustered threats and imposed directives, degrading appraisals of feminized partners, and moralizing formulations such as “You deserve it because you are a filthy dirty slut” that instantiate pious femmephobia. Across the three clusters identified (i.e., commodified femininity, rape-themed fantasies, and dominant top identities) hegemonic masculinity provides the normative ideal of “real men,” femmephobia organizes the devaluation of those who occupy the social location of “woman”, and technology facilitated sexual violence is enacted when these ideals are scripted in profile texts as both desirable and routine.

### Sexual Harm Scripts and Rape Culture Logics in Men Who Have Sex with Men in Digital Spaces

Sexual script theory distinguishes between cultural scenarios, interpersonal scripts, and intrapsychic scripts (Simon & Gagnon, 1986). The *Scruff* profiles sit at the intersection of these levels. They are intrapsychic fantasies rendered public in a tightly constrained digital format, yet they draw heavily on cultural scenarios about what sex “is” and who is expected to do what to whom, and they sketch out interpersonal scripts for future encounters.

Across all three themes, the underlying structure resonates with the normative heterosexual script described in feminist and scripting literatures (Krahé & Berger, 2021; Schippers, 2007), in which an active, desiring partner initiates and drives the encounter while a receptive partner accommodates, yields, and expresses gratitude. In this dataset, however, that structure is reworked through specifically gay top/bottom frameworks rather than simply replaying a male/female binary. In the “use me as you please” profiles, authors describe themselves as passive, receptive, and “made” for penetration, while casting their partners as “men” or “alpha” figures who decide what happens; in the “rape me without mercy” and “I rape you, kick you out of the car, and leave you dead in the gutter” themes, the same asymmetry is intensified through explicit references to rape, being forced, being “bred” without warning, or being beaten and discarded. This does not imply that bottom or feminized sexual roles are inherently unagentic; work on gay male sexual subjectivities shows that bottoming can be experienced as powerful, pleasurable, and chosen, but it does highlight that, in this particular subcorpus, bottom coded positions are repeatedly scripted as violable, disposable, and less entitled to set limits.

These findings extend rape culture research that has largely focused on heterosexual men’s violence against women (Jordan, 2022; Kessel, 2022) by suggesting that rape culture logics can also be taken up and re signified in MSM digital contexts. In line with Jordan’s (2022) account of rape culture as a system that normalizes male sexual entitlement and renders feminized bodies violable, one structurally oriented reading of these profiles is that they redeploy those logics within same sex encounters: some men are cast in a masculinized, agentic role that echoes the “man” position in heteronormative scripts, while others adopt feminized, receptive positions that borrow from, but do not simply reproduce, the “woman” category. The repeated insistence that one “never says no,” that partners should “not ask for permission,” or that “breeding without warning” is what arouses the author suggests that, at the level of textual fantasy, the suspension of consent and the devaluation of feminized partners are presented as desirable elements of these sexual harm scripts. At the same time, alternative readings that foreground consensual kink, camp excess, or ironic play remain possible and are acknowledged below.

The results echo and extend Dietzel’s (2021, 2024) analysis of rape culture on Grindr, which shows how unwanted sexual advances and entitlement are routinized in MSM app interactions. Where Dietzel focuses primarily on interactional exchanges and clickable consent, this study brings attention to how rape themed desire is pre scripted in profile texts themselves, before any interaction occurs. In this aggression-themed subcorpus, the high frequencies of threats and imposed directives in the rape fantasy and dominant top subsets are consistent with Dietzel’s argument that, in some MSM app contexts, digitally mediated interactions can facilitate the normalization of sexual aggression, while also supporting Patel and Roesch’s (2022) broader claim that technology facilitated sexual violence must be understood at the level of everyday communicative practices, not only in extreme or criminal cases.

At the same time, the profiles contribute to debates about how gay men’s sexual practices and risk are framed, including what is often termed “gay male promiscuity.” Klesse (2016) critiques how non-monogamous and highly sexual gay men are pathologized through what he terms the “spectre of promiscuity.” In this corpus, authors explicitly adopt highly sex-focused self-presentations, describing themselves as insatiable, as ‘throwaway cunts’, or as ‘holes’ for many partners, while simultaneously embedding these self-descriptions in rape-themed scripts. This supports McKie et al.’s (2019) governmentality-based account of how gay men’s risk practices are shaped by internalized norms of masculinity and self-governance: here, risk is not only about condomless sex or partner number, but about the internalization of coercive sexual scripts as aspirational.

Framed this way, the concept of sexual harm scripts builds on Wright’s (2011) account of media driven sexual socialization and Bogetić’s (2021) analysis of how dating apps reproduce “locally and globally current normative restraints” around sexuality. Rather than treating aggressive profile content as exceptional, the present study suggests that these scripts form part of a broader cultural repertoire that connects everyday app use with the structural dynamics of rape culture (Jordan, 2022; Tranchese, 2023). In line with Hearn et al. (2025), who argue for “interconnecting violences,” the *Scruff* profiles illustrate how interpersonal sexual aggression is entangled with wider systems of gendered devaluation and control.

### Femmephobia, Masculinities, and the Social Location of “Woman”

The three themes also highlight the central role of femmephobia, the systemic devaluation of femininity, in organizing gendered power in this *Scruff* context (Hoskin, 2017, 2019, 2024). In the feminized receptive and rape fantasy profiles, authors routinely describe themselves through feminized labels (“submissive female”), female coded body parts (“cunt,” “clit”), and conventionally feminine clothing. They present themselves as insatiable, “almost virgins,” or “throwaway” partners who are there to be “used,” “bred,” or “destroyed.”

At the same time, these feminized positions are marked as low status. User and imagined partners refer to the feminized figure as a “slut,” a “bitch,” or a “throwaway slut,” and in some examples explicitly state that they “deserve” to be bred or punished because of this status. This double move, hypersexualising feminized subjects while treating them as stupid, disposable, or contemptible, echoes Hoskin’s (2017) typology of femmephobia, particularly femme mystification, in which femininity is commodified and eroticized while its full humanness is denied. The profiles exemplify the dynamics Hoskin (2017, pp. 102–103) describes: femininity becomes a fetishized object (“a hungry cunt,” “a walking vagina”), detached from personhood and available for masculine use.

These patterns resonate with broader empirical work showing that masculine traits are valorized while femininity is marginalized across contexts (Allan, 2025; Bhatti, 2022). McKie et al. (2019) and Menzie (2022) both note how men who fail to conform to hegemonic masculine ideals are feminized and excluded; in this dataset, that feminization is enacted linguistically through slurs (“faggot,” “bitch,” “slut”) and through the positioning of some MSM as “real women” only by virtue of being penetrated or “bred.” The dominant top profiles, in particular, reproduce a binary between “real men” and feminized others, in which the latter are legitimate targets of aggression and contempt.

At the same time, the results complicate a simple victim perpetrator dichotomy. Many researchers actively seek out feminized positions (“submissive female looking for an alpha man”) and eroticize their own availability and disposability. Drawing on Messerschmidt’s (2019), accounts of plural and changing masculinities, and on gender performative perspectives (Paechter, 2006), these practices can be read as attempts to inhabit hybrid positions that combine elements of masculinity and femininity. However, in this aggression themed corpus, the pleasure attached to feminized positions is almost always bound to subordination, punishment, and self degradation. Feminized roles are desirable precisely insofar as they are violable. This supports Hoskin’s (2019) argument that femmephobia is not only an external stigma but also an internal regulatory mechanism within LGBTQ+ communities (Hoskin et al., 2024). It also aligns with work on “mascing” and networked masculinities in MSM app cultures (Ward, 2016; Roig-Mora et al., 2025), which documents how masculine self-presentation is rewarded while femininity is penalized. In the present study, occupying the social location of “woman” (Hoskin, 2017) is both a resource, a way of attracting “alpha” partners, and a liability, a position associated with disposability and reduced entitlement to safety. As Hearn et al. (2025) suggest, these patterned devaluations of femininity are not incidental; they are central to how gendered violence is organized and justified.

### Negotiating Consent, Fantasy, and Technology-Facilitated Sexual Violence

A key tension in the corpus concerns how consent is imagined and where harm is located. Many of the scenarios described could, in principle, form part of consensual role play: they involve explicit role differentiation, detailed descriptions of rough practices, and a clear eroticization of power imbalance. In keeping with the coding procedures, we therefore do not treat rape themed content as direct evidence of non-consensual behavior; instead, we distinguish between the presence of a rape themed fantasy frame and the specific utterances within that frame that discursively suspend or relocate consent, for example “I never say no” or “Do not ask for permission.” From this perspective, the analytic focus is not on determining whether any given encounter would in practice be consensual, but on how the profile texts themselves erase or trivialize mechanisms that are emphasized to safeguard consent, such as prior negotiation, agreed signals or safe words, and the right to withdraw.

Instead, men in the dataset frequently define desirability precisely through the absence of these safeguards. They describe themselves as never saying no, instruct partners not to ask for permission, and invite being “raped,” “drilled,” or “bred without warning.” On the dominant top side, authors state that they will not stop “until I’m done,” that they will “stomp” on a partner’s neck “whenever I feel like it,” or that the partner should not even make contact if they “can’t take it.” The quantitative distribution of discourse acts reflects this: in the rape fantasy and dominant top subsets, threats and impositions of a course of action are far more frequent than in the broader feminized receptive profiles, while elicitations focused on mutual negotiation are comparatively rare.

These findings complement Dietzel’s (2024) work on “clickable consent” among MSM, which shows that consent on apps is often treated as something that can be given once and for all, or implied by profile content, rather than as an ongoing, revisable process. Where Dietzel focuses primarily on how users understand and practice consent in online and offline interactions, this study highlights how, in an aggression focused corpus, consent is pre emptively waived discursively: profiles script the self as someone who never refuses, does not want partners to ask for permission, and is excited by being taken without warning.

From the perspective of technology-facilitated sexual violence (Patel & Roesch, 2022), this matters because dating apps do not simply connect partners; they also shape vocabularies of desire and can contribute to making particular risk configurations appear routine or unremarkable. Fileborn (2016) and Colliver (2023) both show that young adults and MSM on apps manage safety within ambiguous and often unsafe environments, where norms of risk and responsibility are contested. This study adds a discursive layer to that work by showing that, within this aggression themed subcorpus, many users articulate sexual subjectivities in which their own boundaries are presumed to be permanently relaxed or irrelevant.

The recurrent framing of aggression as ordinary or desirable in these profiles also resonates with content analyses of mainstream pornography, which document the high prevalence of verbal and physical aggression, especially toward women (Fritz et al., 2020; McKee, 2015). While the *Scruff* data are user generated and do not allow direct inference about offline behavior, the recurrence of threats, imposed acts, and degrading appraisals suggests that pornographic repertoires of aggression and degradation have been appropriated into everyday sexual self-presentation, now applied to feminized MSM rather than to women. In line with Mustanski et al. (2011), who show how relationship characteristics and sexual scripts shape risk taking among young MSM, the present findings point to the need to think of “risk” not only in terms of condom use or partner number but also in terms of the routinization of scripts that render consent negotiable and pain or humiliation desirable.

It is important not to collapse all rape themed fantasy into actual violence or to pathologize consensual kink practices that may be carefully negotiated and meaningful to participants. Following Fiske’s (1987) account of polysemy and Plummer’s (2002) work on sexual storytelling, these profile texts can, in principle, sustain multiple readings (i.e., dominant, negotiated, and oppositional), depending on readers’ positions and investments. The present analysis foregrounds a structurally oriented, “dominant” reading that focuses on how the profiles normalize particular distributions of power and vulnerability, while acknowledging that participants themselves may experience these scenarios as playful exaggeration, cathartic fantasy, or subcultural in jokes. The profiles do not provide access to offline dynamics or to subjective experiences. However, they do show that certain ways of talking about sex, what we have termed sexual harm scripts, are publicly rehearsed and circulated in this MSM digital space. Given existing evidence that MSM experience and perpetrate sexual harm in complex ways (Colliver, 2023; Scheer et al., 2021), the repeated circulation of these scripts in this aggression-focused subcorpus may have implications for how sexual coercion is recognized, named, or minimized.

### Sexual Subjectivity, Agency, and the Limits of Resistance

Finally, the findings raise questions about sexual subjectivity and agency in MSM app based cultures. On one reading, in line with work on gay male sexual subjectivities and erotic agency (e.g., Plummer, 2002), the men who describe themselves as insatiable, “throwaway cunts,” or “submissive females” are not simply subject to an external script; they are actively performing particular identities, experimenting with taboo fantasies, and pursuing specific forms of pleasure. Similarly, those who present themselves as “alpha fuckers” who “breed” or “punish” others may experience these as stylized, playful performances rather than literal promises, and as ways of enacting a relational gender dynamic in which they respond to partners’ expressed or presumed desires for a more masculinized top.

However, as Connell (1995, 2003) and Schippers (2007) remind us, agency is always exercized within patterned constraints, and as McKie et al. (2019) show, gay men’s sexual practices are shaped by subtle forms of self-governance linked to hegemonic masculinity. The fact that different authors converge on remarkably similar formulations, never saying no, being bred without warning, tearing someone apart, treating partners as “sluts” or “bitches” who “deserve” punishment, indicates that these are shared cultural repertoires rather than idiosyncratic fantasies. Even when such scripts are embraced, they may still carry costs: they reinforce the marginalization of effeminate men, devalue care and reciprocity in sex, and risk obscuring the line between desired roughness and unwanted harm.

The aggression focused nature of the subcorpus necessarily limits what can be seen. Other work on MSM apps documents more reciprocal, playful, or explicitly resistant scripts (Byron & Albury, 2018; Gerena & Pilkay, 2025; Steele et al., 2024). Outside the present dataset, MSM clearly use digital platforms to create counter spaces, build community, and contest body and gender norms. Nonetheless, within this purposive subcorpus of profiles that explicitly reference sexual aggression, sexual harm scripts recur frequently and are tightly bound to negotiations of gendered position, power, and pleasure.

Interpreting these patterns through sexual script theory (Simon & Gagnon, 1986) and the literatures on hegemonic and plural masculinities (Connell, 1987, 1995, 2003; Connell & Messerschmidt, 2005) and femmephobia (Blair & Hoskin, 2015; Hoskin, 2017, 2019, 2024; Hoskin et al., 2024) allows us to see MSM not as lying outside rape culture logics, but as actively participating in their reproduction, adaptation, and eroticization. This does not exhaust the range of MSM sexual possibilities, but it does identify a powerful cluster of scripts that shape how sex is imagined, proposed, and justified in a specific app based context. In line with Hearn et al. (2025), these findings underscore the importance of understanding how everyday digital practices, such as writing a profile, interconnect with broader violences, including the devaluation of femininity, the ways in which sexual coercion can be normalized, and the constraining of sexual agency for those who occupy feminized positions.

### Limitations

The study’s design imposes clear limits on what can be claimed. The analytic dataset consists of 300 *Scruff* profiles from a single metropolitan region (Madrid), all authored by MSM aged 40–50 and all explicitly referencing sexual aggression. This is a purposive sub-corpus constructed to enable in-depth analysis of aggression-themed content, not a representative sample of *Scruff* users or of MSM more broadly. As a result, the findings cannot be used to estimate the prevalence of rape-themed fantasies or sexual aggression on *Scruff*, nor can they be straightforwardly generalized to younger or older men, to other regions or countries, or to users of different platforms. The data capture self-presentations at a single point in time and do not include interactional exchanges or offline encounters; they tell us how users choose to describe themselves and their desires, not what happens in practice. These constraints do not undermine the value of the findings but mean that the results are best understood as offering analytical rather than statistical generalization: they identify mechanisms and patterns that may travel to other MSM digital contexts, but they require empirical testing elsewhere.

Building on these insights, future research should pursue at least three directions. Comparative studies across platforms (e.g., *Scruff*, *Grindr*, and other apps) and across cohorts (younger and older MSM, as well as trans and non-binary users) are needed to map how sexual harm scripts and femmephobia vary with platform affordances, age, and intersectional positionings. Multi-method designs that combine profile analysis with interviews, focus groups, or digital ethnography would help to clarify how users themselves understand rape-themed fantasies, how they negotiate consent and boundaries in practice, and to what extent online scripts shape face-to-face encounters. Research in offline contexts such as bars, sex venues, and community spaces could also explore whether and how the norms rehearsed on apps bleed into broader sexual cultures among MSM, and where there is resistance, refusal, or reworking of these scripts. Such work would not only deepen theoretical debates about queer masculinities, femmephobia, and sexual scripts, but also inform the design of interventions that aim to foster safer, more equitable intimate cultures in both online and offline worlds. Even with these caveats, the study advances understanding of how femmephobia, hegemonic masculinities and sexual harm scripts are reproduced and contested in MSM dating profiles, and it points to clear directions for theoretical refinement and prevention‑oriented research.

## Conclusion

The present study has examined how sexual scripts are articulated and negotiated in *Scruff* profiles authored by Spanish men who have sex with men (MSM) aged 40–50, focusing specifically on an aggression-themed sub-corpus. Through a mixed-methods discourse-analytic approach, we have shown that users recurrently script themselves and others through feminized self-presentations that commodify femininity, rape-themed fantasies that present male sexual entitlement as central to desire, and dominant “top” identities that presume consent and celebrate control. Taken together, these patterns suggest that rape-culture logics are not external to MSM digital spaces but are actively reproduced, reworked, and eroticized through femmephobic sexual harm scripts in this online environment.

Theoretically, the study extends sexual script theory and the literature on rape culture by foregrounding MSM as not only implicated in, but also directly affected by, technology-facilitated sexual violence. By tracing how users strategically occupy the social position of “woman” while casting their partners as “men,” the analysis highlights the co-constitution of masculinities and femininities within same-sex male encounters. In this context, femmephobia (i.e., the systematic devaluation of femininity) emerges as a central mechanism through which gendered power is organized, even in spaces where no women are present. We argue that the concept of sexual harm scripts, understood as patterns within cultural and interpersonal sexual scripts that normalize, excuse, or trivialize coercive practices, offers a productive way of linking individual fantasies and profile descriptions to broader cultural scenarios of rape culture. The data suggest that MSM are not simply exempt from gendered logics of sexual violence; rather, they re-enact and re-signify those logics through the feminization and disposability of certain positions within the MSM community.

Methodologically, this research contributes to work on technology-facilitated sexual violence by combining qualitative discourse analysis with a functional coding of discourse acts adapted from Tsui’s (1994) model. Treating *Scruff* profile texts as sequences of eliciting, directive, and informative acts makes it possible to see sexual harm scripts not merely as “themes” but as patterned ways of doing things with words: inviting, demanding, threatening, appraising, or trivializing aggression. This approach offers a replicable framework for analyzing user-generated sexual content that is sensitive to both micro-linguistic detail and macro-level gendered power relations. In addition, the focus on a non-Anglophone, middle-aged cohort of MSM, together with a carefully documented translation and coding procedure, broadens a literature that has often centered younger users, Anglophone contexts, and more general samples of profiles.

The findings also have important practical implications. They suggest that sexual health and consent education directed at MSM cannot focus solely on individual risk behaviors (such as condom use or partner number) but should explicitly address femmephobia, the eroticization of coercion, and the ways in which rape-themed fantasies can be positioned as desirable markers of sexual desirability and masculinity in some MSM digital contexts. Consent-focused interventions should create space to differentiate between consensual kink and the uncritical reproduction of scripts that present refusal as negotiable, pain as proof of desire, or feminized partners as less entitled to safety. The analysis also points to a need for collaboration with dating-app providers to design platform-level responses that make it easier to flag, challenge, or contextualize violent sexual content; without pathologizing consensual fantasy. This might include clearer community standards, optional content warnings, or in-app resources about consent and boundaries tailored to MSM. Practitioners and support services working with MSM who have experienced sexual harm should likewise be aware that violence may be narrated, and sometimes minimized, through these sexual harm scripts; understanding this discursive context can inform assessment, prevention, and therapeutic work.

## Footnotes

1 Here we understand physical aggression as “any action that clearly could reasonably be expected to cause physical harm to oneself or another person” (Fritz et al., 2020, p. 3045), and verbal aggression as “any action that clearly could reasonably be expected to cause psychological harms to oneself or another person, regardless of the perpetrator’s intent and the target’s response” (Fritz et al., 2020, pp. 3045–3046). In this light, all the profiles selected include examples of verbal aggression such as name-calling (e.g., looking for a filthy little bitch); suggestions of aggression in the profile’s name (e.g., merciless brutal huge cock); and/or aggressive acts that describe both consensual and non-consensual sexual aggression (e.g., I slap buts, bite nipples. I’ll fuck you bareback, slap your ass till I come no matter how much you whine).
